# Incidence and Characteristics of Multiple Primary Cancers: A 20-Year Retrospective Study of a Single Cancer Center in Korea

**DOI:** 10.3390/cancers16132346

**Published:** 2024-06-26

**Authors:** Jin-Hee Kwon, Heyjin Kim, Jin Kyung Lee, Young Jun Hong, Hye Jin Kang, Yoon Jung Jang

**Affiliations:** 1 Medical Science Demonstration Center, Korea Cancer Center Hospital, Korea Institute of Radiological and Medical Sciences, Seoul 01812, Republic of Korea; peterjane19@kirams.re.kr; 2 Department of Laboratory Medicine, Korea Cancer Center Hospital, Korea Institute of Radiological and Medical Sciences, Seoul 01812, Republic of Korea; jklee@kirams.re.kr (J.K.L.); clinchem@kirams.re.kr (Y.J.H.); 3 Division of Hematology-Oncology, Department of Internal Medicine, Korea Cancer Center Hospital, Seoul 01812, Republic of Korea; hyejin@kirams.re.kr (H.J.K.); dbswjd0305@kirams.re.kr (Y.J.J.)

**Keywords:** multiple primary cancer, second primary cancer, cancer survivors

## Abstract

**Simple Summary:**

The incidence of multiple primary cancers (MPCs) among 96,174 cancer patients at the Korea Cancer Center Hospital (KCCH) from 2003 to 2022 was 2.3%. Breast cancer (15.7%) was the most common primary cancer, with lung cancer (15.2%) being the most frequent second primary cancer. The median latency period for second primary cancers was 4.1 years, decreasing for third and fourth primary cancers (2.1 years and 1.6 years, respectively). Lymphoma had the highest relative risk (2.1) for developing MPCs. Specific primary cancers showed significant associations with subsequent cancers, informing future research and management strategies.

**Abstract:**

Rising cancer survival rates have led to an increased risk of multiple primary cancers (MPCs). Data on MPCs in South Korea are limited. This study aimed to address incidence and clinical characteristics of MPCs in a single cancer center in Korea during a 20-year period. We retrospectively analyzed 96,174 cancer patients at the Korea Cancer Center Hospital between 2003 and 2022, identifying 2167 patients with metachronous MPCs based on Surveillance, Epidemiology, and End Results SEER criteria. We categorized patients by cancer type (15 major solid cancer groups and 3 major hematologic cancer groups), including pathological diagnosis, assessed latency periods, and relative risks (RRs) for developing MPCs. The overall MPC incidence was 2.3%. Breast cancer (15.7%) was the most common primary cancer, and lung cancer (15.2%) was the most frequent second primary cancer. The median latency period for second primary cancers was 4.1 years. Decreasing latency periods for third and fourth primary cancers were observed (2.1 years and 1.6 years, respectively). Most cancers maintained their dominant pathological type despite notable changes in the prevalence of specific pathologies for certain types of second primaries. Lymphoma showed the highest RR (2.1) for developing MPCs. Significant associations were found between specific primary and subsequent cancers, including breast–ovary, thyroid–breast, stomach–pancreas, colorectal–head and neck, lung–prostate, and lymphoma–myeloid neoplasms. These findings contribute to a better understanding of MPC occurrence. They can inform future research on their etiology and development of improved management strategies.

## 1. Introduction

The landscape of cancer care is evolving rapidly, with advancements in diagnosis and treatment leading to a significant increase in cancer survivors worldwide [1]. Many cancer survivors face an increased risk of developing a new, completely different, non-metastatic cancer after diagnosis of the first primary cancer, which is known as a multiple primary cancer (MPC). MPCs are estimated to occur in 2–17% of cancer survivors, representing a 1.1- to 1.6-fold greater risk compared to the general population [2]. This rising trend necessitates further research to understand and manage MPCs effectively.

Although the exact mechanisms behind MPC development remain unclear, several factors are suspected to contribute. Early detection advancements may lead to the identification of concurrent or closely following cancers. Increased life expectancy allows more time for additional cancers to emerge. Additionally, age at first cancer diagnosis, the specific cancer type, and its stage at diagnosis influence the risk of developing an MPC. Notably, preceding therapeutic interventions, particularly DNA-damaging treatments such as radiation therapy and chemotherapy administered for the initial cancer, have been demonstrated to elevate the risk of subsequent MPCs. Genetic predisposition, environmental exposures, and lifestyle habits such as smoking and alcohol consumption also play potential roles [2].

Recent studies across various regions have pinpointed specific cancer types with higher risks of developing subsequent MPCs. Swedish research has found that oral cavity/pharynx, Hodgkin lymphoma, laryngeal, and esophageal cancers can elevate MPC risk [3]. Australian data have revealed a higher relative risk (RR) of MPC in men after melanoma and in women after head and neck cancers [4]. Additionally, a UK study has identified an increased risk for individuals under 50 who have previously experienced breast or melanoma cancers [5].

Despite growing global research interest, nationwide data on MPCs in South Korea remain scarce. Existing studies primarily focus on the incidence and survival of second malignant neoplasms in childhood cancer survivors, with limited data available for the adult population [6,7]. This study addresses this gap by representing the largest investigation of MPCs in Korean adults to date. We retrospectively analyzed demographic and clinical characteristics of an MPC cohort at a single cancer center over a 20-year period. Specifically, we investigated the association between primary cancer site and the development of subsequent primaries. Our findings provide preliminary data to inform future research on the etiology and treatment of MPCs, ultimately leading to improved healthcare for this expanding patient population.

## 2. Materials and Methods

### 2.1. Data Source

Data for cancer patients were obtained from Electronic Medical Records (EMRs) of the Korea Cancer Center Hospital (KCCH) from 2003 to 2022. Among them, patients with cancer codes assigned were selected to establish an overall cancer patient group. Cancer codes were assigned according to the 8th edition of the Korean Standard Classification of Diseases (KCD). KCD adheres to the International Classification of Diseases, 10th revision (ICD-10) format. Patient information, including registration number, initial cancer diagnosis date, age at diagnosis, and histopathological examination results (including bone marrow studies), was verified through individual EMR charts.

### 2.2. Patient Cohort

This study focused on patients aged 19 years or older who were diagnosed with two or more completely different cancers. The definition of MPCs was based on the criteria defined by the Surveillance, Epidemiology, and End Results (SEER) in the U.S. In the data screening process, eligible patients were defined according to KCD and KCCH data classification settings. MPC patients were determined based on the following criteria: (1) patients with two or more distinct KCD cancer codes recorded for more than two months and (2) patients diagnosed with the same cancer code after two months but different histopathological findings. Solid cancers were determined based on pathologic diagnosis. Hematologic cancers were determined based on bone marrow diagnosis. Tumor registries like SEER and other studies on MPCs have collected data on both malignant tumors and carcinoma in situ. Consequently, cases of carcinoma in situ were also included in the MPC group [8,9]. Cases where in situ cancers progressed to carcinomas were excluded. Exclusion criteria were: (1) those who received another code within two months (considered as synchronous cancers); (2) those with newly assigned cancer code being metastatic or recurrent cancer; (3) those with insufficient clinical evidence or records for the diagnosed cancer code through EMR chart review. To simplify statistical analysis, this study classified cancers into 18 major categories: 15 solid cancer categories (head and neck, esophagus, stomach, colorectal, liver/bile duct/gallbladder, pancreas, lung, bone and soft tissue, skin, breast, uterus and cervix, ovary, prostate, kidney/urinary tract/bladder, and thyroid) and 3 hematologic cancer categories (lymphoma, plasma cell neoplasms, and myeloid neoplasms (MNs)). MNs were identified using both specific ICD-10 codes (C91–C95) and codes for myelodysplastic neoplasms (MDS) and myeloproliferative neoplasms (MPN) (D45–D47). All other cancers were categorized as “others”. Pathological classifications for each cancer type categorized pathologies with less than 5 cases as “others”. Pathologies included in “others”, “rare epithelial carcinomas” of breast cancer, and “other leukemias” are challenging to classify. They are listed in Table S4.

**Figure 1:**
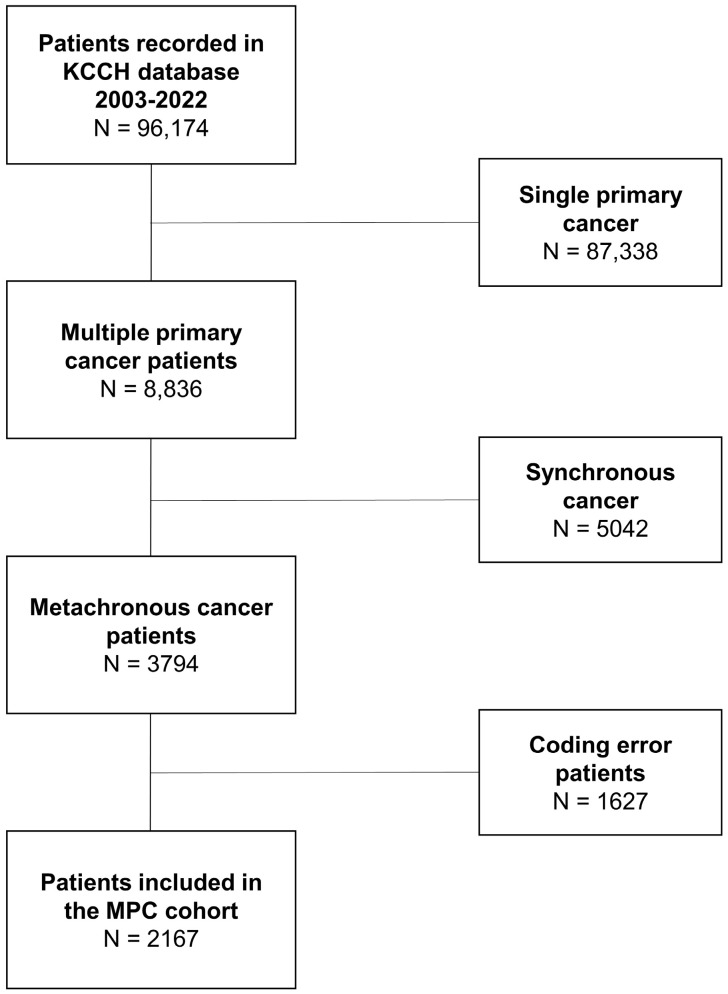
Flowchart showing the selection of patients with multiple primary cancers.

A total of 96,174 adult cancer patients diagnosed with their first cancer at KCCH between 2003 and 2022 were included in this study. Of these, 87,338 had a single primary cancer diagnosis, while 8836 (9% of all cancer patients) had two or more cancer codes. These patients were classified according to the SEER criteria into those diagnosed with two cancer codes within two months (synchronous cancer patients: n = 5042) and those not diagnosed (metachronous cancer patients: n = 3794). To ensure accuracy, a comprehensive review for code errors was conducted. This included examining for duplicate C-codes, C-codes for metastasis, and insufficient evidence for corresponding pathological C-codes. The review also included analysis of pathological diagnoses and clinical charts via electronic medical records (EMRs) for the 3794 metachronous cancer patients. As a result, 1672 patients were excluded from the MPC study cohort due to code errors. The remaining 2167 patients comprised the final MPC study cohort (Figure 1).

### 2.3. Statistical Analysis

All statistical analyses were conducted using Excel program ver. Microsoft office Professional Plus 2019 (Microsoft Co., Redmond, WA, USA) and IBM SPSS Statistics ver. 21.0 (IBM Co., Armonk, NY, USA). We compared patients with first primary cancer and those with MPCs using *t*-test and chi-square test in SPSS, calculating *p*-values, RRs with 95% confidence intervals, and generating forest plots using Excel program ver. Microsoft office Professional Plus 2019. Both SPSS and Excel were used to calculate cancer type frequencies.

## 3. Results

### 3.1. Characteristics of the Study Cohort

Table 1 summarizes characteristics of the entire research cohort. From 2003 to 2022, a total of 96,174 adult cancer patients were registered at KCCH, with 2.2% (2167) developing metachronous MPCs. The proportion of subsequent cancers increased significantly after the second primary, reaching 11.3% (26 of 231) for fourth primary cancers. The overall gender distribution favored females (56.9%) for first primary cancers. Males made up a higher proportion of patients with second (54.6%) and third (51.1%) primary cancers. However, females outnumbered them for fourth primary cancers (53.9%). The median age increased with subsequent primary cancers from 57 years for the first to 63 years for the second and 67 years for both third and fourth. MPC diagnoses increased over time. The highest number of diagnoses for first primary cancer occurred between 2003 and 2007 (36.4%), although the peak shifted to 2018–2022.

### 3.2. Sites of Multiple Primary Cancers

**Figure 2:**
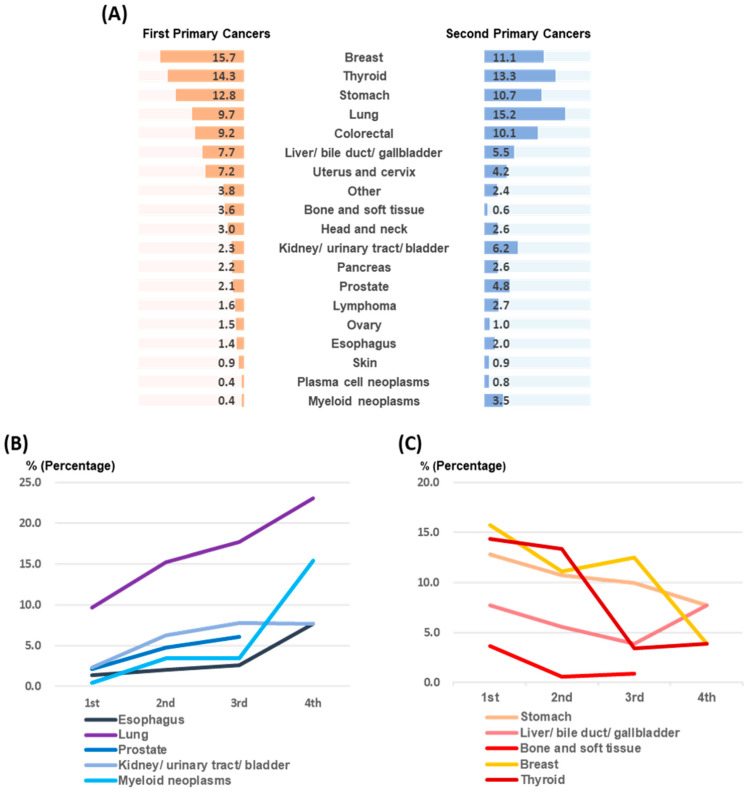
Incidence of first and multiple primary cancers: (**A**) Distribution of first primary cancer of all cancer patients and second primary cancer of MPC patients. (**B**) Cancers showing significant increasing trends. (**C**) Cancers showing significant decreasing trends.

Figure 2 presents overall distribution of cancer sites for both first primary and second primary cancers and trends of subsequent primary cancers. Breast, thyroid, stomach, lung, and colorectal cancers comprised the top five first primary cancers (15.7%, 14.3%, 12.8%, 9.7%, and 9.2%, respectively). This dominance continued for second primaries, with lung, thyroid, breast, stomach, and colorectal cancers again featuring prominently (15.2%, 13.3%, 11.1%, 10.7%, and 10.1%, respectively) (Figure 2A). Lung cancer was more frequent for second primary cancer in males (20.3%), while breast cancer remained dominant in females (20.4%) (Figure S1). Figure 2B,C highlight significant increasing or decreasing trends (*p* < 0.05) observed over time. Subsequent primary cancers revealed increasing rates for lung, skin, prostate, and kidney/urinary tract/bladder cancers and MNs. Conversely, stomach, liver/bile duct/gallbladder, bone/soft tissue, breast, and thyroid cancers exhibited decreasing trends. Data for in situ cancers (D codes) were incorporated (Table S1 and Figure S2). MNs demonstrated the highest D code proportions for both first (47%) and third (40%) primary cancers, while uterus/cervix cancers had the highest proportion for second primary cancers (75%).

### 3.3. Latency Period of Multiple Primary Cancers

**Figure 3:**
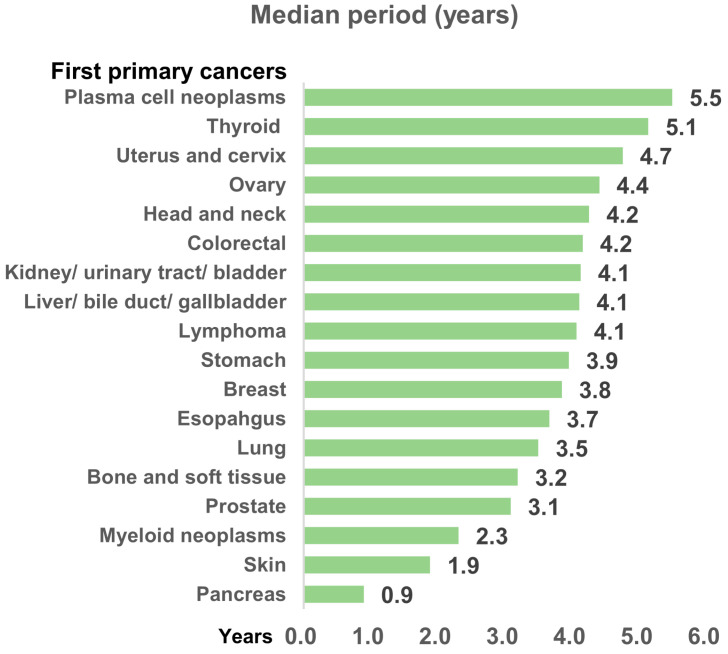
Median period (years) to multiple primary cancers by type of first primary cancer.

Analysis of latency periods revealed that 44% of patients with MPCs were diagnosed with second primary cancers within 1–5 years after the first. This trend continued for subsequent primary cancers, with 40% of third primary cancers and 42% of fourth primary cancers being diagnosed within 1–5 years of the preceding primary cancer. A decreasing trend in latency period was observed for subsequent primary cancers, with the median interval period between diagnoses being 4.1 years for first to second primary cancers, 2.1 years for second to third (*p* = 0.000), and 1.6 years for third to fourth (*p* = 0.963) (Table 2). Figure 3 presents latency periods by first primary cancer type. Plasma cell neoplasms exhibited the longest delay (5.5 years), while pancreatic cancer had the shortest latency period (0.9 years). Complete distribution of median latency periods and patient numbers for specific second primary cancers based on first cancer type are shown in Tables S2 and S3.

### 3.4. Second Primary Cancer Distribution According to Age at First Primary Cancer Diagnosis

In the age group of 19–49 years, the most common second primary cancer is thyroid cancer (24.3%), followed by breast cancer (23.1%) and uterus/cervix cancer (8.2%). For those aged 50–59 years, lung cancer (16.0%) and thyroid cancer (14.8%) are predominant, with breast cancer also being significant (12.1%). In the 60–69-year age group, lung cancer remains the most frequent (19.3%), followed by colorectal cancer (CRC) (11.7%) and stomach cancer (11.4%). Among individuals aged 70–79 years, lung cancer continues to be prevalent (20.9%), with stomach cancer (14.8%) and CRC (11.5%) also notable. Finally, in the over-80-year age group, stomach cancer (16.1%), lung cancer (16.1%), and prostate cancer (16.1%) are the leading second primary cancers (Table 3).

### 3.5. Pathologic Patterns in Multiple Primary Cancers

**Figure 4:**
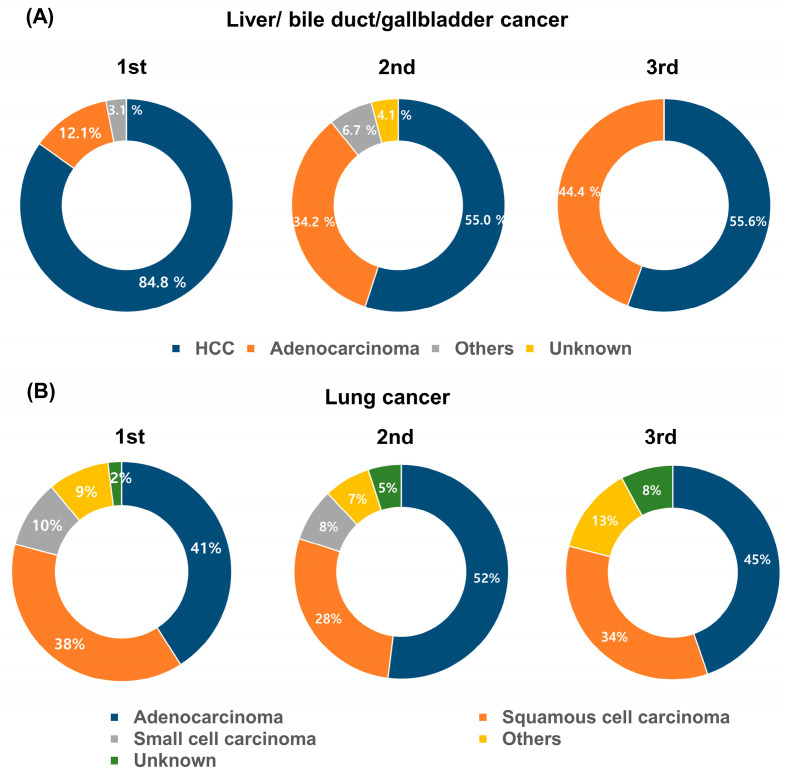
Pathologic diagnosis of first, second, and third primary cancers: (**A**) Pathologic types of liver/bile duct/gallbladder cancer. (**B**) Pathologic types of lung cancer. Abbreviations: HCC, hepatocellular carcinoma.

Although most cancers maintained their dominant pathology in subsequent primary cancers, notable shifts emerged for rare types: mucoepidermoid carcinoma (head and neck), GIST (stomach), neuroendocrine carcinoma (colorectal), other types (bone and soft tissue, breast, uterus and cervix, kidney/urinary tract/bladder), basal cell carcinoma (skin), and AML (MNs) (Table S4). Adenocarcinoma in liver, bile duct, and gallbladder significantly increased in second primary cancers (Figure 4A), while incidence of squamous carcinoma in cervix and follicular carcinoma in thyroid of second primary cancers significantly decreased or disappeared. Moreover, in the case of lung cancer, when comparing dominant types of adenocarcinoma and squamous cell carcinoma, their proportions were similar in the first primary cancer (41.5% and 38.2%, respectively). In the second primary cancer, there was a significant increase in the proportion of adenocarcinoma (52.3%), while squamous cell carcinoma decreased to 27.7% (*p* = 0.028) (Figure 4B).

### 3.6. Relative Risk for Multiple Primary Cancers

**Figure 5:**
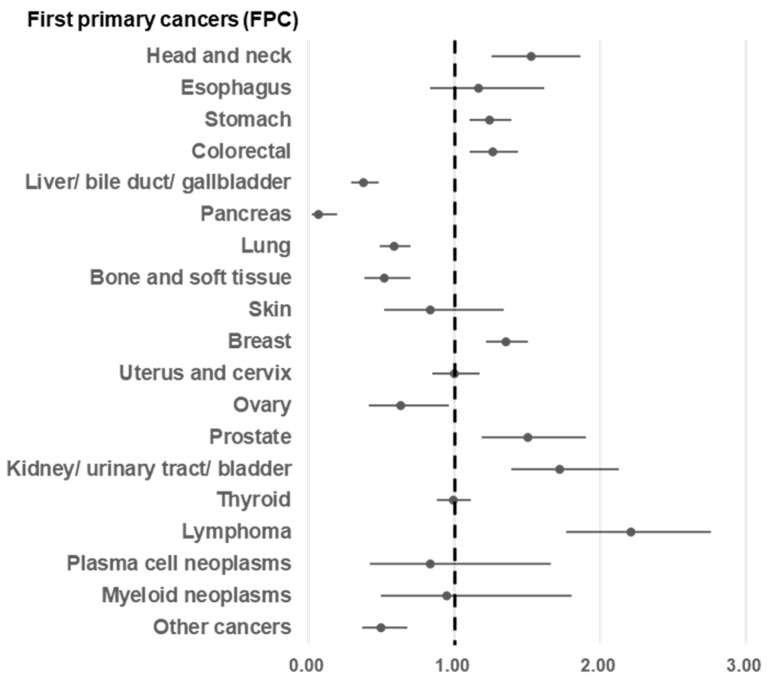
Relative risk of multiple primary cancers by type of first primary cancer.

**Figure 6:**
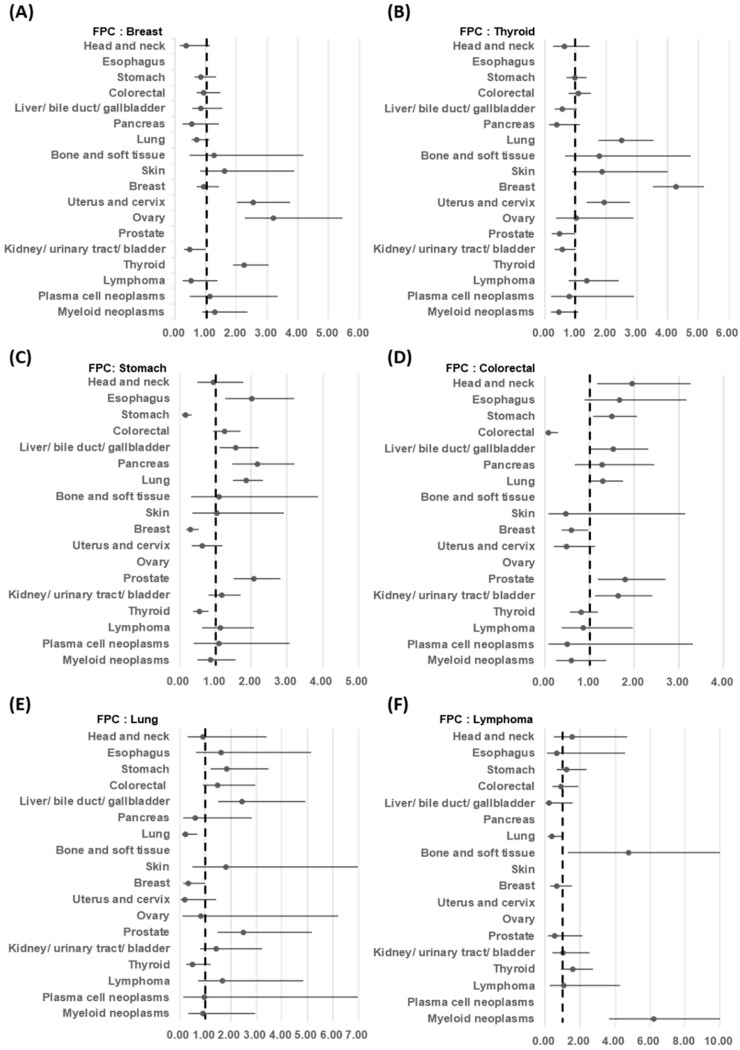
Relative risks for specific second primary cancers among patients with first primary breast cancer (**A**), thyroid cancer (**B**), stomach cancer (**C**), colorectal cancer (**D**), lung cancer (**E**), and lymphoma (**F**).

Analysis revealed an increased risk of developing subsequent primary cancers for patients with first primary cancers (Figure 5, Table S5): head and neck cancer (RR = 1.53, 95% CI = 1.25 to 1.86), esophagus cancer (RR = 1.16, 95% CI = 0.84 to 1.62), stomach cancer (RR = 1.26, 95% CI = 1.10 to 1.39), colorectal cancer (RR = 1.26, 95% CI = 1.10 to 1.44), breast cancer (RR = 1.35, 95% CI = 1.22 to 1.50), prostate cancer (RR = 1.50, 95% CI = 1.19 to 1.90), kidney/ urinary tract/ bladder cancer (RR = 1.72, 95% CI = 1.39 to 2.13), and lymphoma (RR = 2.21, 95% CI = 1.77 to 2.76). Figure 6 and Table S6 provide further details on the risk of developing specific second primary cancers after first primary cancers of the breast, thyroid, stomach, colon or rectum, or lung or lymphoma. The highest RRs were observed for ovarian cancer after breast cancer (RR = 3.19, 95% CI = 2.26 to 4.51), breast cancer after thyroid cancer (RR = 4.27, 95% CI = 3.53 to 5.19), pancreatic cancer after stomach cancer (RR = 2.16, 95% CI = 1.46 to 3.21), head and neck cancer after CRC (RR = 1.95, 95% CI = 1.16 to 3.27), prostate cancer after lung cancer (RR = 2.48, 95% CI = 1.48 to 4.17), and MNs after lymphoma (RR = 6.20, 95% CI = 3.64 to 10.55).

## 4. Discussion

**Figure 7:**
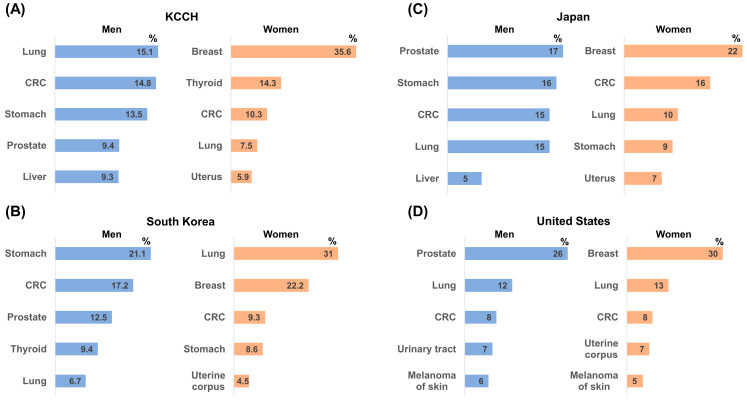
Comparison of cancer prevalence in 2021: (**A**) KCCH; (**B**) South Korea [11]; (**C**) Japan [12]; (**D**) United States [13].

This study analyzed data from a single cancer center to investigate the prevalence and characteristics of MPCs. It was found that 2.3% of patients with first primary cancer developed MPCs, emphasizing the need for ongoing surveillance. However, this figure likely underestimates the true prevalence due to the lack of access to data from other hospitals and death records. Common cancer types observed at the center were generally in line with global and domestic cancer statistics (Figure 7) [1,10,11,12,13]. Notably, the prevalent cancer types for both men and women at the center aligned with patterns observed in Asian countries. When comparing South Korea, China, and Japan, lung cancer and stomach cancer emerged as the most prevalent types for men, while breast cancer, stomach cancer, and CRC were the most commonly occurring cancers for women [9,10,11]. However, national differences in incidence rates were observed for specific cancers, particularly prostate, thyroid, and skin cancers [14], with the prostate cancer incidence rate being lower than in Western countries for males, while the incidence rate of thyroid cancer was slightly higher than that of breast cancer in females. Skin cancer rates were significantly lower in Korea than in Western countries, potentially due to inherent factors such as skin photo-type and sun exposure habits [15]. Understanding these differences in MPC prevalence and specific cancer types across populations can inform screening programs and diagnostic strategies for high-risk individuals. Our study identified lung, thyroid, breast, stomach, and colorectal cancers as the most common MPCs, aligning with observations in prior European research [16]. A distinct discrepancy emerged for thyroid cancer, where we observed a higher prevalence compared to other studies. This difference could be attributed to two factors: (1) the specific patient population at KCCH, which tended to have a higher proportion of breast and thyroid cancer patients compared to other institutions, and (2) the generally highest incidence rate of thyroid cancer in South Korea [17]. The median time between diagnoses of the first and second primary cancers was 4.1 years, consistent with research findings in the United States [18]. A trend of decreasing latency period between primary and subsequent cancers suggests potential shared risk factors or biological mechanisms. The latency period of MPCs can vary depending on the type of cancer, individual characteristics, and treatment modalities. In our cohort, the shortest latency period (0.9 years) was observed for patients with first pancreatic cancer and the longest latency period (5.5 years) was observed for those with first plasma cell neoplasms, similar to findings of previous studies [19,20].

We confirmed that second primary cancers occurred similarly in different ages, but there were some differences (Table 3). Specifically, second primary gastrointestinal (GI) tract cancers (stomach cancer and CRC) exhibited a higher prevalence in older groups (60–79 and over 80) compared to younger groups (19–49 and 50–59) diagnosed with a first cancer. This aligns with the established knowledge of increasing GI tract cancer risk with age, and a similar trend is observed for second primary colorectal cancer (spCRC) in older adults. An analysis using SEER data found a higher incidence of spCRC, especially in those aged 65 or older. Interestingly, despite an overall decline in spCRC incidence between 2000 and 2016, older adults remained the majority of diagnosed cases [21]. Consistent with our findings of a heightened incidence of second primary prostate cancer in individuals aged 70 and older, a previous study documented that over 67% of patients diagnosed with this condition were aged 65 or above [22]. Furthermore, we observed an increased incidence of second primary kidney, urinary tract, and bladder cancers in patients aged 70 or older. This might be connected to the elevated relative risk (4.95; 95% CI: 2.93–8.27) of these specific cancers in our KCCH prostate cancer population, which represents the highest risk compared to other second primary cancers. Notably, prostate cancer itself primarily affects older adults, with the highest incidence reported in men aged 75–79 [23]. This suggests a potential association between the late onset of second primary kidney, urinary tract, and bladder cancers with a late-stage first primary prostate cancer. The treatment course for pelvic cancers can influence the subsequent development of kidney, urinary tract, and bladder cancers. A study investigating patients who received RT for pelvic cancers reported a hazard ratio (HR) for bladder cancer of 3.48 (95% CI: 2.94–4.12) for the 50–69 age group and 4.25 (95% CI: 3.56–5.07) for the 70–84 age group, highlighting a heightened risk in those aged 70 and above [24]. A recent study found no significant difference in the risk of developing second primary cancers between patients receiving surgery or RT for prostate cancer. However, the risk of developing second primary genitourinary cancers was significantly higher in the RT group, with an adjusted subhazard ratio (aSHR) of 2.29 (95% CI: 1.16–4.51) [25]. These observed patterns underscore the importance of implementing age-specific surveillance and preventive strategies. The disproportionately higher incidence of specific second primary cancers in older age groups suggests that cancer survivors could benefit from personalized follow-up care plans that consider both their age and the type of primary cancer.

Pathological type can affect the choice of treatment methods and prognosis. Although most subsequent primary cancers maintained the dominant pathology of their initial counterpart (Table S4), notable shifts in prevalence of specific pathologies were observed for 12 second primary cancer types, including head and neck cancer, stomach cancer, colorectal cancer, liver/bile duct/gallbladder cancer, lung cancer, bone and soft tissue cancer, skin cancer, breast cancer, uterus and cervix cancer, kidney/urinary tract/bladder cancer, thyroid cancer, and MNs. In our cohort, second primary lung cancers exhibited a significant increase in adenocarcinoma (52.3%) and a significant decrease in squamous cell carcinoma (27.3%) (*p* = 0.028) (Figure 4B). Previous research has suggested that smoking influences the prevalence of histological types in primary lung cancer [26]. Additionally, treatment modalities for the first primary cancer can affect the distribution of histological types in subsequent cases. For instance, patients receiving radiation therapy had a higher proportion of adenocarcinomas, while those undergoing surgery only had a higher proportion of squamous cell carcinomas [27]. These findings imply that both smoking and treatment modalities play potential roles in shaping the histological type of lung cancer, both in primary and secondary occurrences. Our study confirmed a significant association between first primary lung cancer and subsequent prostate cancer, as reported in prior research studies [28,29]. The risk varied depending on the lung cancer subtype, with adenocarcinoma patients having the highest risk for prostate cancer, consistent with a multi-country study [29]. It is important to note that the histological type of lung cancer can vary widely within each group of patients. Individual characteristics such as age, gender, and genetic factors can also play a role in determining the histological type of lung cancer.

This study aligned with previous research studies by highlighting elevated risks for specific subsequent primary cancers (MPCs) following specific initial diagnoses at KCCH. Significant associations between specific primary and subsequent cancers were revealed in our cohort, including associations between breast and ovary cancers (breast–ovary), thyroid–breast, stomach–pancreas, colorectal–head and neck, lung–prostate, and lymphoma–MNs. These intricate associations likely stem from a multifaceted interplay of factors, including inherited genetic predisposition, treatment-related genetic damage, age, and other individual risk profiles. The study of genetic predisposition has significantly impacted cancer research, leading to the identification of over 100 cancer predisposition genes [30]. A study found that 21% of MPC patients had pathogenic variants, and among them, 44% carried high-penetrance genes (e.g., *BRCA1/2*, *TP53*, mismatch repair genes) [31]. While our study did not assess cancer predisposition gene (CPG) status, the observed associations between first and second primary cancers suggest a potential role for CPG in contributing to the observed patterns. Ovarian cancer emerged as the most likely MPC among those with first primary breast cancer, supported by numerous studies and the established genetic link, especially in *BRCA1/2* mutation carriers [32,33]. In line with other research, KCCH data revealed an increased risk of subsequent breast cancer after thyroid cancer. Shared factors such as hormone receptors are likely to contribute to this association [34]. Further evidence suggests a potential genetic link between thyroid cancer and breast cancer, with studies in Poland showing a four-fold increase in thyroid cancer risk following a breast cancer diagnosis, rising to a nine-fold increase in women carrying a *CHEK2* mutation [35]. Pancreatic cancer was the most likely MPC after stomach cancer, aligning with a US population-based study [36]. Research has suggested a potential link between these cancers and *BRCA2* mutations, influencing risk, survival, and age of onset [37]. Additionally, several hereditary cancer syndromes are related to gastric and pancreatic cancer, including Lynch syndrome, hereditary diffuse gastric cancer syndrome, familial adenomatous polyposis, and hereditary pancreatic cancer. These syndromes involve inherited genetic defects in mismatch repair genes *CDH1*, *APC*, *BRCA 1/2*, or *ATM*, respectively [38]. Colorectal cancer exhibited a significantly increased risk of developing head and neck cancers, confirming previous findings. A cohort study in Taiwan has found an increased standardized incidence ratio (SIR) of 1.34 for head and neck cancer in this population [39]. While a direct genetic link for this specific cancer pairing is currently lacking, further investigation is warranted. The primary cause of lung cancer is smoking. Additionally, studies have investigated the link between smoking and the risk of prostate cancer, showing that heavy smokers and former smokers have an increased risk of developing prostate cancer [40]. These findings suggest that smoking could be a contributing factor to both lung cancer and prostate cancer, although direct genetic links between the two are less well established. The highest risk of developing subsequent primary cancers was revealed in patients with first primary lymphoma (RR: 2.21). Similar to other studies [41,42], lymphoma patients exhibited the highest risk for developing MPCs, particularly MNs. Swedish data showed a five-fold increase in MDS/AML and a 1.4-fold increase in solid cancers following non-Hodgkin lymphoma (NHL) [41]. Additionally, a cohort study has demonstrated a high SIR (12.87) for therapy-related MNs after lymphoma, suggesting treatment as a potential risk factor [43]. Studies examining the relationship between lymphoma and MNs in Italy have shown that survivors of NHL treated with chemotherapy and monoclonal antibodies have an increased risk of developing second primary malignancies, including therapy-related myeloid neoplasms (t-MNs) [33]. Moreover, the germline *DDX41* mutation has been linked to both acute myeloid leukemia/myelodysplastic syndrome and B-cell lymphoma, further highlighting the complex genetic interactions involved [44]. These findings contribute to a greater understanding of high-risk cancer combinations and can inform the development of more effective prevention strategies. Further research is needed to elucidate the underlying mechanisms driving these associations and to identify potential interventions to mitigate risk.

Although this study was conducted using a relatively small-scale cohort within a single cancer center, the findings of this study largely mirrored nation-level and international trends observed in both overall cancer patients and those with MPCs. In addition, obtaining detailed data such as previous anticancer therapy, medical histories, and lifestyle habits crucial for understanding risk factors was challenging from the EMR chart review of our cohort. There is particularly concerning evidence of increased lung cancer risk in irradiated breast cancer patients, as well as the well-established link between cytotoxic therapies and the development of MNs [45]. Furthermore, this study acknowledges limitations in analyzing family history and genetic information, particularly cancer predisposition genes. A large cohort study has estimated that up to 8% of cancer patients harbor predisposition genes [46]. A recent US study of 1580 MPC patients has revealed a concerning rate of 21% with pathogenic variants [31]. This highlights the potential significance of genetic factors in MPCs. Further research exploring these factors in the Korean population is warranted. Unfortunately, incomplete death records within our cohort hindered comprehensive survival analysis. Several studies have also reported similar obstacles in acquiring comprehensive records, including death records and other cancer records diagnosed at previous or different hospitals, for cancer patients [47]. These studies underscore the challenges in obtaining reliable mortality data, which in turn affects the analysis of statistical outcomes. Similarly, we faced difficulties acquiring complete death and cancer diagnosis records from other hospitals for our entire cohort. This hindered a thorough assessment of follow-up time for survival analysis and accurate estimation of MPC incidence. As a result, our findings on the incidence of MPC patients among cancer patients should be interpreted with caution, considering the potential impact of incomplete mortality data on our analysis. To improve the accuracy of future studies, it is crucial to establish more reliable and comprehensive death record databases, enabling researchers to better understand the long-term outcomes of cancer patients and the factors contributing to their mortality. Previous studies have reported poorer prognoses for MPC patients, particularly when considering molecular characteristics of a subsequent cancer and comorbidities associated with the initial malignancy or underlying illness requiring cytotoxic therapy [48].

This study underscores the importance of tailored surveillance strategies for patients with specific cancer histories, considering the potential for developing MPCs. Further research is crucial to elucidate the underlying mechanisms driving this association and develop targeted prevention and early detection approaches.

## 5. Conclusions

In conclusion, our study provided data on characteristics of MPC patients in a single cancer center. Despite the limited scale of the cohort in a single cancer center, the study’s results closely paralleled national and global trends of common cancer types and incidence of multiple primary cancers. Based on relative risk analysis, specific associations were noted between first primary cancers and subsequent primary cancers, such as breast–ovary, thyroid–breast, stomach–pancreas, colorectal–head and neck, lung–prostate, and lymphoma–myeloid neoplasms. However, further studies considering patients’ treatment history, survival rates, and epidemiological data such as family history and cancer genetic information are needed for a better understanding of these findings.

## Figures and Tables

**Table 1:** Characteristics of patients.

	First Primary Cancers		Second Primary Cancers		Third Primary Cancers		Fourth Primary Cancers				
	n	%	n	%	n	%	n	%			
Sex											
Males	41,490	43.1	1184	54.6	118	51.1	12	46.2			
Females	54,684	56.9	983	45.4	113	48.9	14	53.8			
Total	96,174		2167		231		26				
Age, Median (range)	57 (19–101)		63 (20–103)		67 (29–92)		67 (40–93)				
Age at diagnosis											
19–49	29,351	30.5	315	14.5	24	10.4	2	7.7			
50–59	24,145	25.1	530	24.5	31	13.4	6	23.1			
60–69	23,732	24.7	612	28.2	82	35.5	7	26.9			
70–79	15,020	15.6	566	26.1	72	31.2	9	34.6			
80 years and over	3926	4.1	144	6.6	22	9.5	2	7.7			
Year of diagnosis											
2003–2007	34,979	36.4	180	8.3	5	2.2	0	0.0			
2008–2012	24,165	25.1	522	24.1	47	20.3	2	7.7			
2013–2017	18,657	19.4	668	30.8	65	28.1	9	34.6			
2018–2022	18,373	19.1	797	36.8	114	49.4	15	57.7			
	**First Primary** **Cancers**		**Second Primary** **Cancers**		**Third Primary** **Cancers**		**Fourth Primary** **Cancers**		***p*-value**		
	**n**	**%**	**n**	**%**	**n**	**%**	**n**	**%**	**1 vs. 2**	**1 vs. 3**	**1 vs. 4**
Type of Primary cancer											
Solid cancer											
Head and neck (C00–14, C30–32)	2924	3	56	2.6	9	3.9	0	0	0.221	0.449	0.367
Esophagus (C15)	1340	1.4	43	2	6	2.6	2	7.7	0.021	0.119	0.006
Stomach (C16, D00)	12,269	12.8	232	10.7	23	10	2	7.7	0.005	0.202	0.439
Colorectal (C18–20, D01)	8806	9.2	218	10.1	19	8.2	2	7.7	0.15	0.624	0.796
Liver/bile duct/gallbladder (C22–24)	7408	7.7	120	5.5	9	3.9	2	7.7		0.03	0.998
Pancreas (C25)	2109	2.2	56	2.6	6	2.6	1	3.9	0.22	0.675	0.565
Lung (C33–34, D02)	9282	9.7	329	15.2	41	17.8	6	23.1			0.02
Bone and soft tissue (C40, C49)	3472	3.6	12	0.6	2	0.9	0	0		0.025	0.324
Skin (C43–44, D04)	902	0.9	19	0.9	9	3.9	0	0	0.77		0.62
Breast (C50, D05)	15,104	15.7	241	11.1	29	12.6	1	3.9		0.189	0.097
Uterus and cervix (C53–54, D06)	6902	7.2	91	4.2	11	4.8	2	7.7		0.155	0.919
Ovary (C56)	1473	1.5	21	1	4	1.7	0	0	0.034	0.805	0.525
Prostate (C61)	2061	2.1	103	4.8	14	6.1	0	0			0.451
Kidney/urinary tract/bladder (C64–68, D09)	2233	2.3	135	6.2	18	7.8	2	7.7			0.069
Thyroid (C73)	13,773	14.3	289	13.3	8	3.5	1	3.9	0.195		0.127
Hematologic cancer											
Lymphoma (C81–85, C88)	1578	1.6	58	2.7	7	3	0	0		0.097	0.51
Plasma cell neoplasms (C90)	426	0.4	18	0.8	1	0.4	0	0	0.008	0.982	0.734
Myeloid neoplasms (C91–95, D45–47)	423	0.4	75	3.5	8	3.5	4	15.4			
Others	3689	3.8	51	2.4	7	3	1	3.9		0.524	0.998
Total	96,174	100	2167	100	231	100	26	100			

**Table 2:** Latency periods of multiple primary cancers.

	First Primary Cancer to Second Primary Cancer		Second Primary Cancer to Third Primary Cancer		Third Primary Cancer to Fourth Primary Cancer		*p*-Value	
							1st to 2nd vs. 2nd to 3rd	2nd to 3rd vs. 3rd to 4th
	n	%	n	%	n	%		
Latency period								
Under 2 months			58	25.1	5	19.2		
2 months to less than 6 months	142	6.6	8	3.5	2	7.7		
6 months to less than 1 year	172	7.9	26	11.3	2	7.7		
1 year to less than 5 years	948	43.8	93	40.3	11	42.3		
5 years to less than 10 years	604	27.9	33	14.3	5	19.2		
Over 10 years	301	13.9	13	5.6	1	3.9		
Median year (range)	4.1 (00–19.9)		2.1 (0–14.1)		1.6 (0–10.3)		0.000	0.963

**Table 3:** Second primary cancer incidence by age at first primary cancer diagnosis.

Age	19–49		50–59		60–69		70–79		Over 80	
	Cancer type	%	Cancer type	%	Cancer type	%	Cancer type	%	Cancer type	%
1	Thyroid	24.3	Lung	16.0	Lung	19.3	Lung	20.9	Stomach	16.1
2	Breast	23.1	Thyroid	14.8	Colorectal	11.7	Stomach	14.8	Colorectal	16.1
3	Uterus and cervix	8.2	Breast	12.1	Stomach	11.4	Colorectal	11.5	Lung	16.1
4	Stomach	7.9	Colorectal	10.6	Thyroid	8.6	Kidney, urinary tract, and bladder	10.5	Prostate	16.1
5	Colorectal	6.7	Stomach	9.8	Liver, bile duct, and gallbladder	8.0	Prostate	8.9	Kidney, urinary tract, and bladder	12.9

## Data Availability

The data presented in this study are available on request from the corresponding author.

## Supplementary Materials

The following supporting information can be downloaded at: https://www.mdpi.com/article/10.3390/cancers16132346/s1, Figure S1: Distribution of first and second primary cancers by gender. (A) Males and (B) females; Figure S2: Distribution of C codes and D codes by type of cancer. (A) First primary cancers. (B) Second primary cancers. (C) Third primary cancers; Table S1: Patient number of latency period categorized by the type of first primary cancer; Table S2: Patient number of latency period categorized by the type of first primary cancers; Table S3: Complete distribution of median latency periods and patient numbers for specific second primary cancers; Table S4: Pathologic distribution by the type of cancer in a cohort of patients with multiple primary cancers; Table S5: Relative risk of multiple primary cancers by type of first primary cancer; Table S6: Relative risk of specific second primary cancers by type of first primary cancer.
