# Supplementary figures and images for: Incidence and Characteristics of Multiple Primary Cancers: A 20-Year Retrospective Study of a Single Cancer Center in Korea
Source: Cancers (Basel). 2024 Jun 26;16(13):2346. doi: 10.3390/cancers16132346 (PMC11240339; doi:10.3390/cancers16132346)

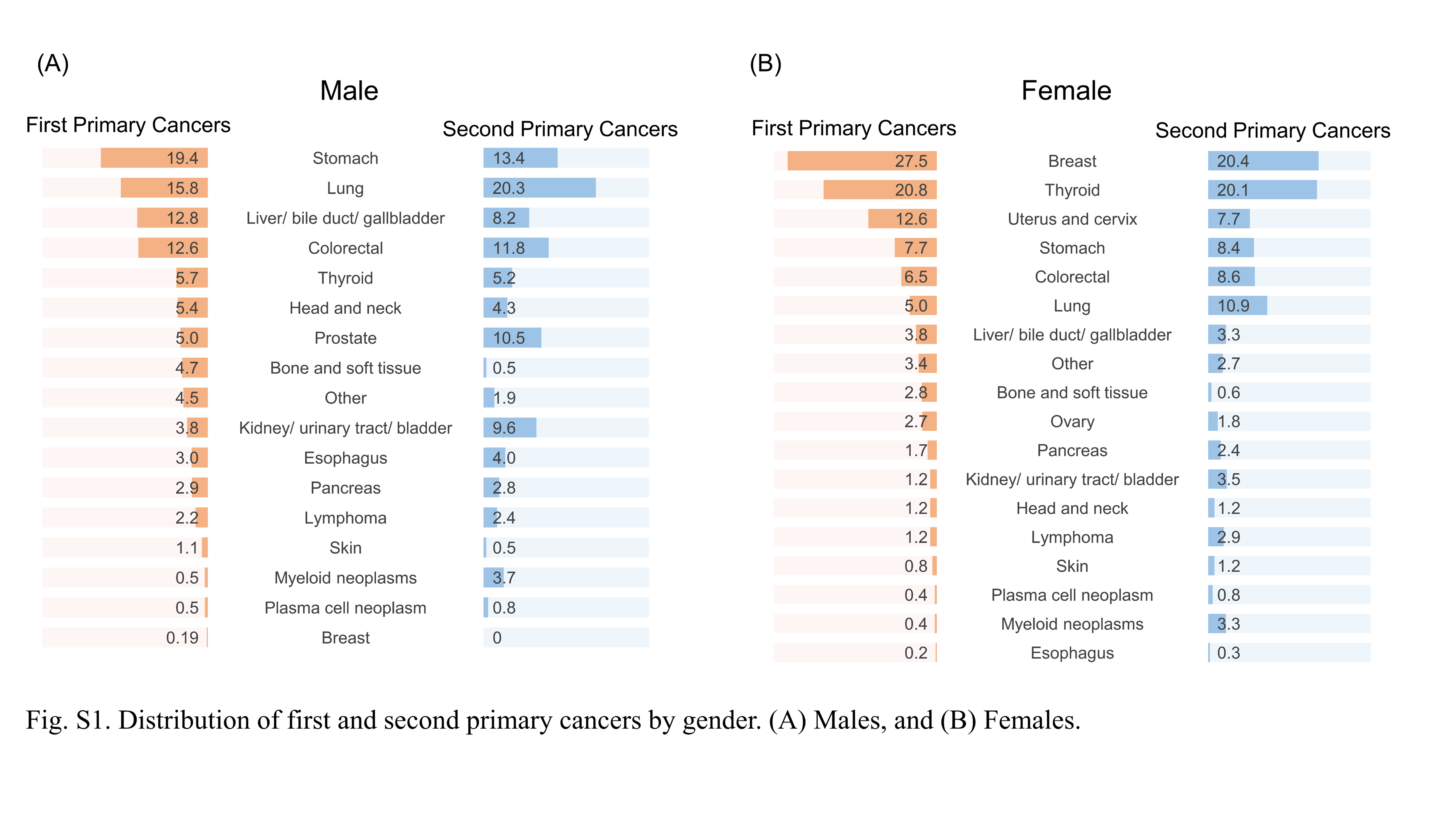

Supplement: Supplementary file 1 [file cancers-16-02346-s001.zip › Figure S1.TIF]

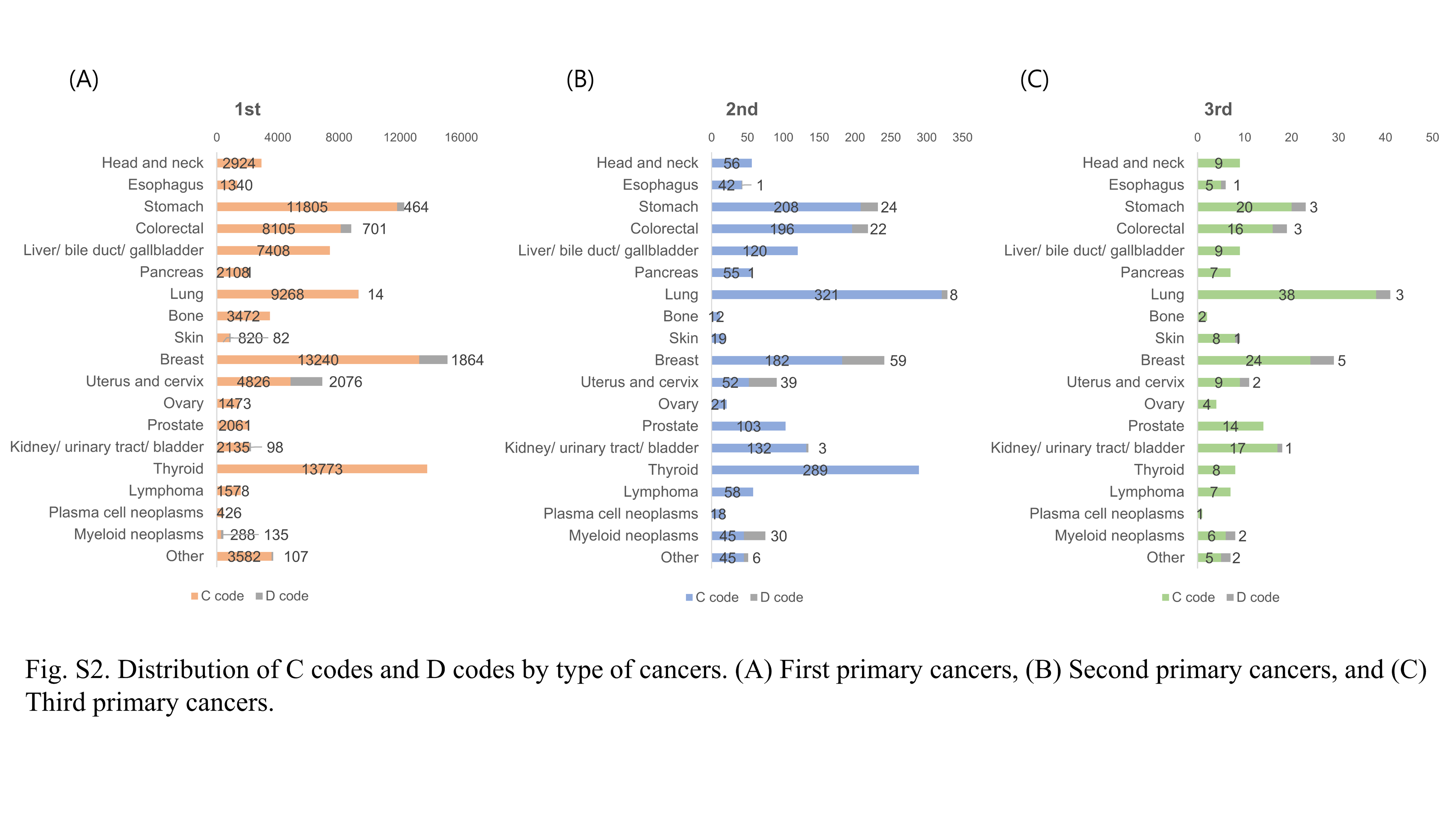

Supplement: Supplementary file 1 [file cancers-16-02346-s001.zip › Figure S2.TIF]

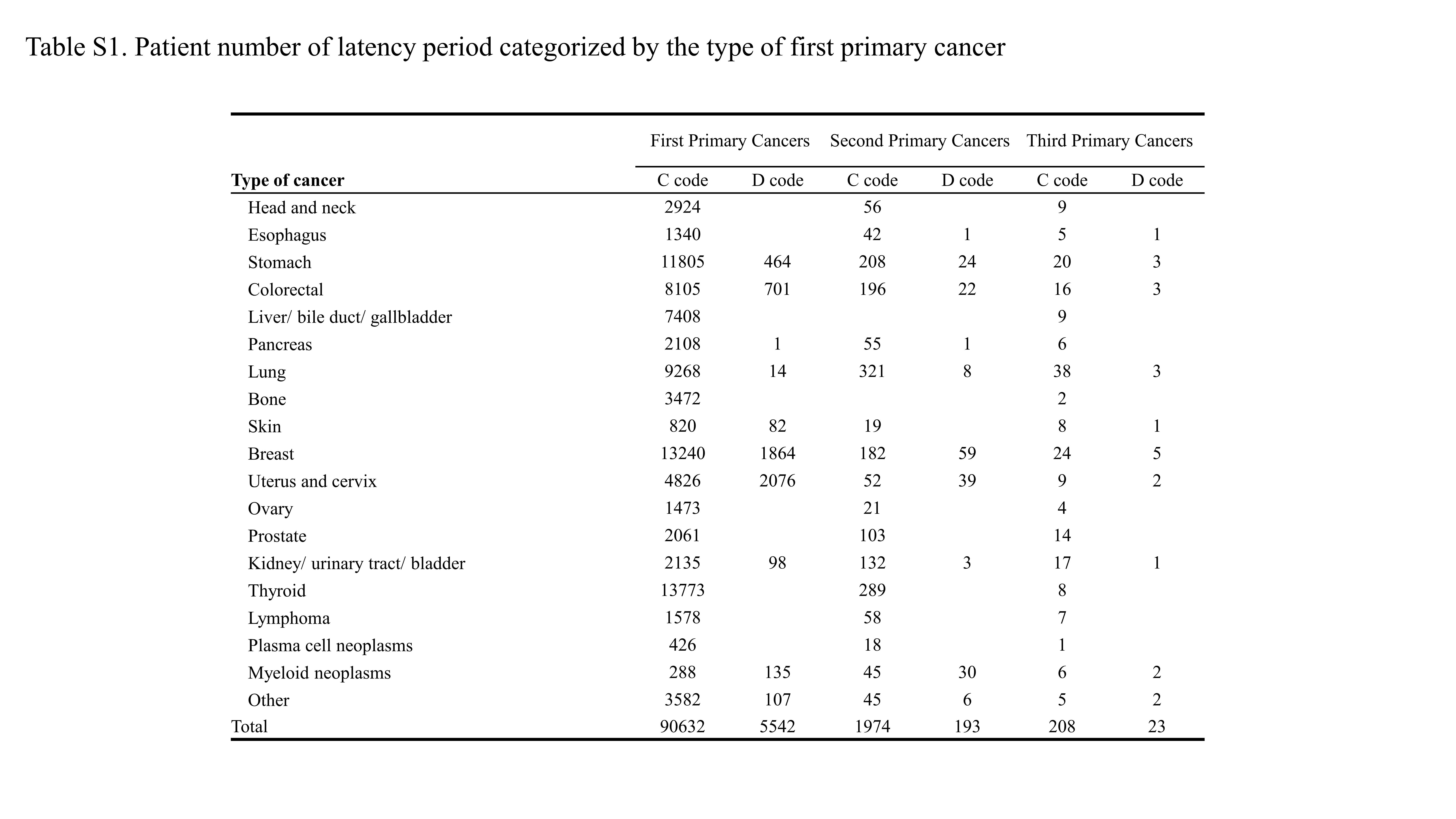

Supplement: Supplementary file 1 [file cancers-16-02346-s001.zip › Table S1.TIF]

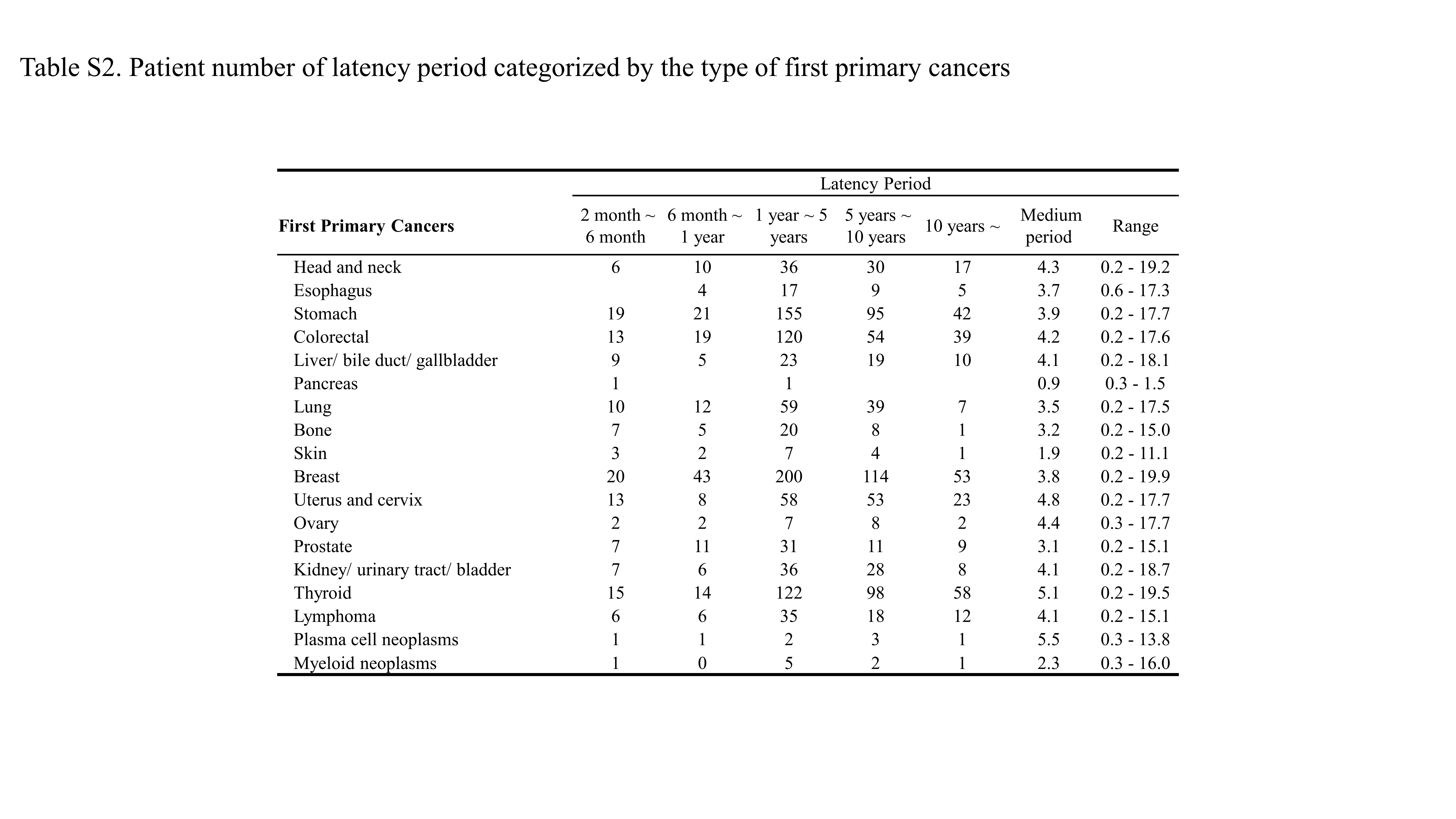

Supplement: Supplementary file 1 [file cancers-16-02346-s001.zip › Table S2.TIF]

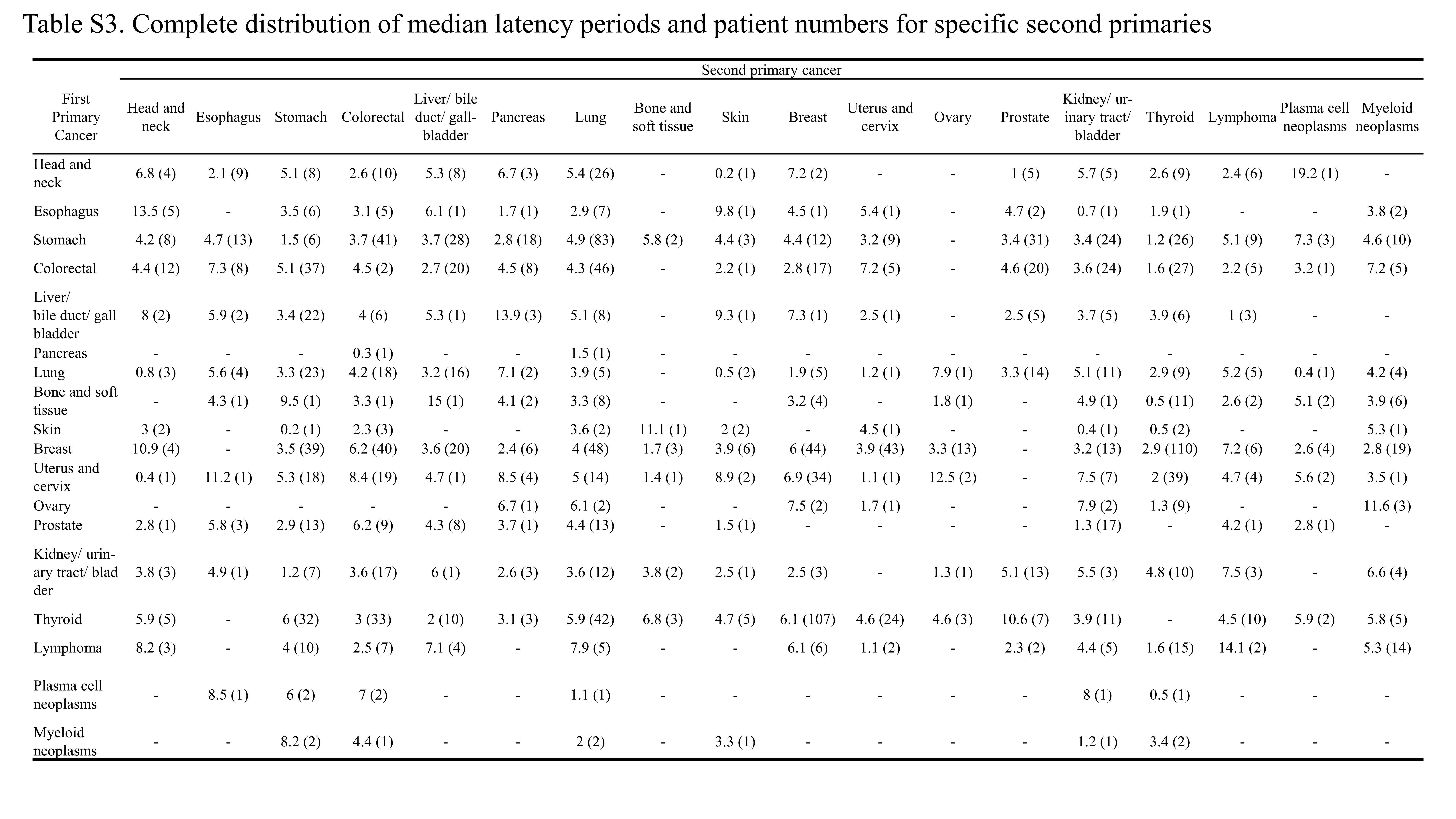

Supplement: Supplementary file 1 [file cancers-16-02346-s001.zip › Table S3.TIF]

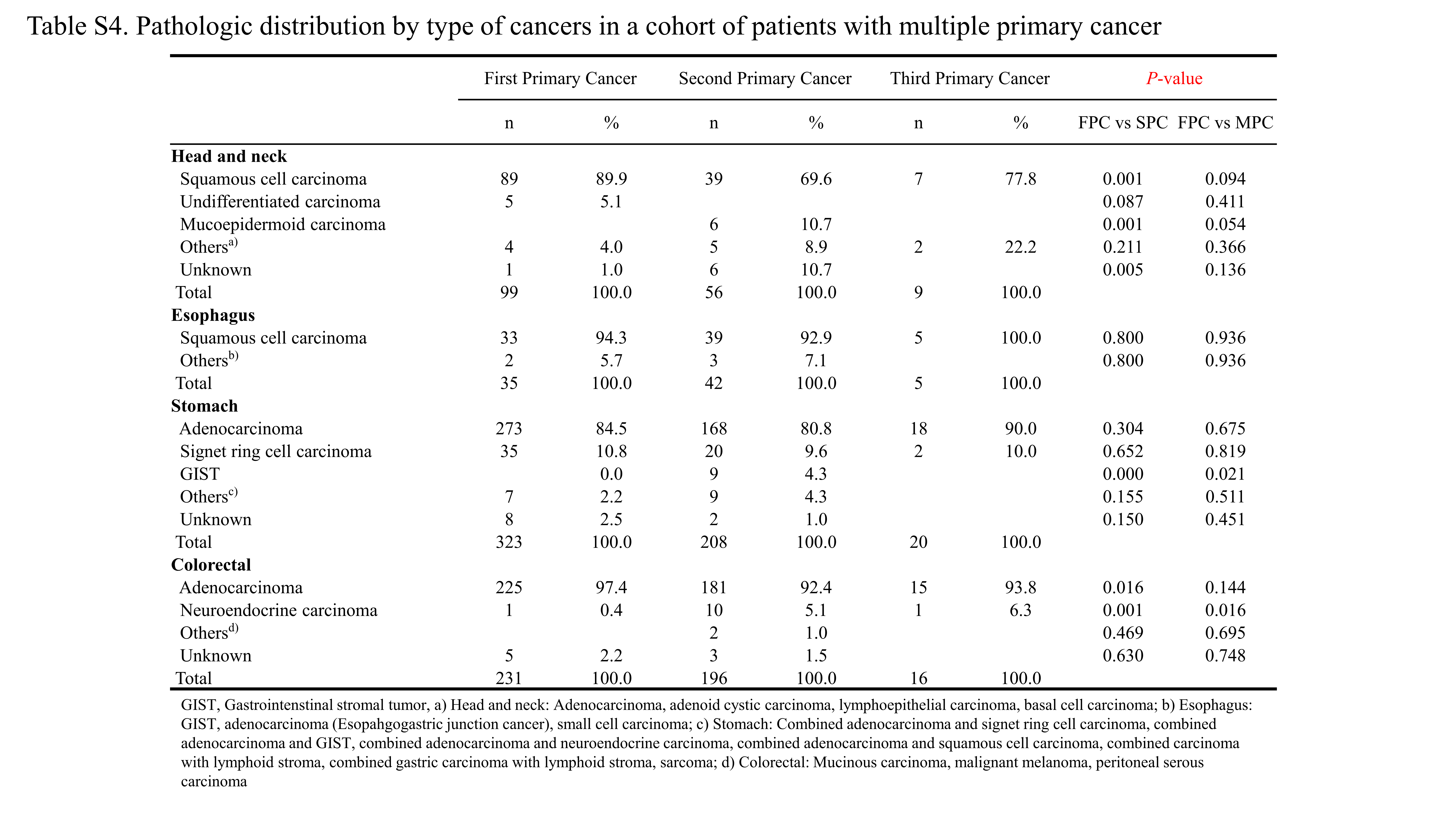

Supplement: Supplementary file 1 [file cancers-16-02346-s001.zip › Table S4-1.TIF]

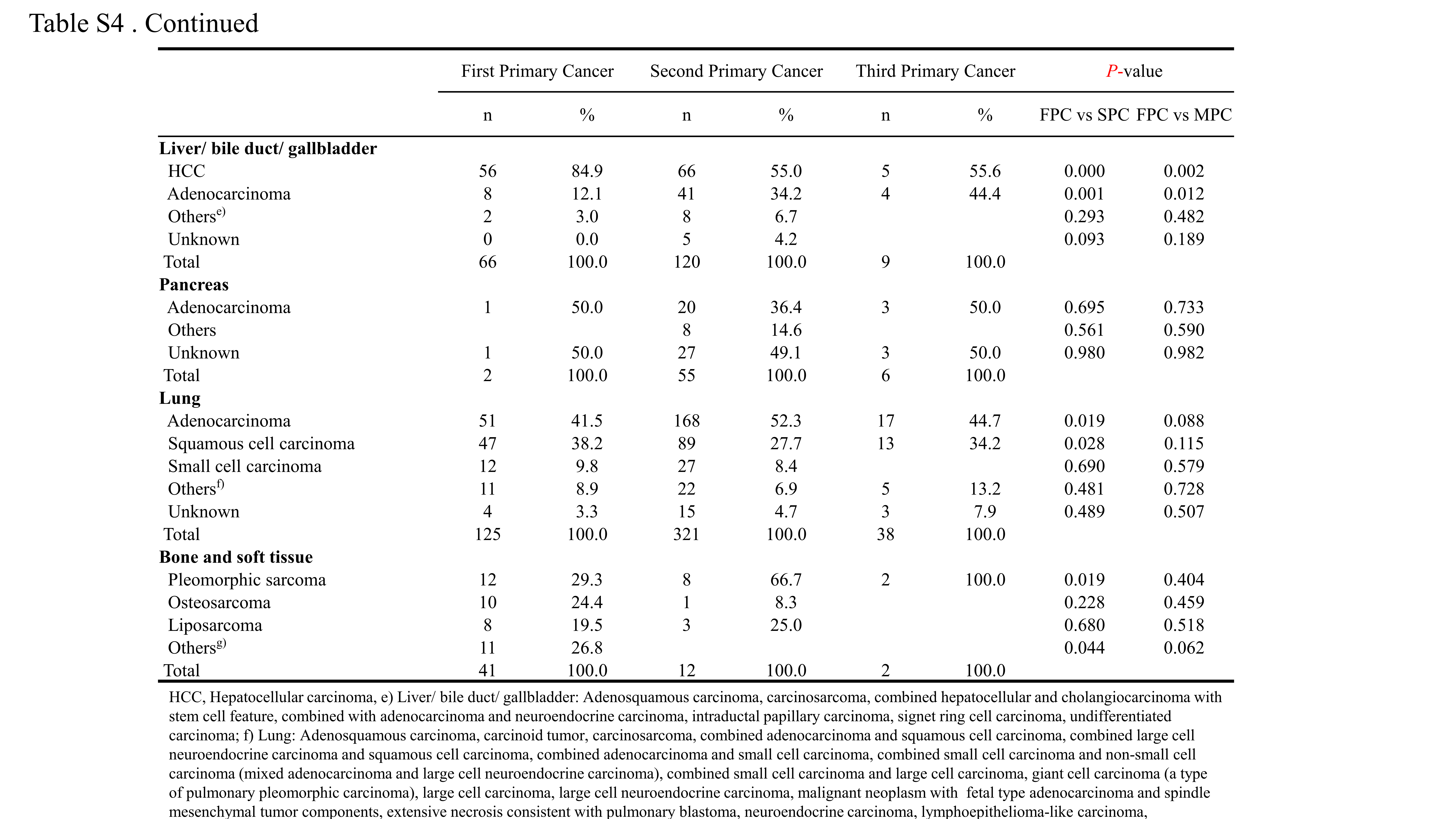

Supplement: Supplementary file 1 [file cancers-16-02346-s001.zip › Table S4-2.TIF]

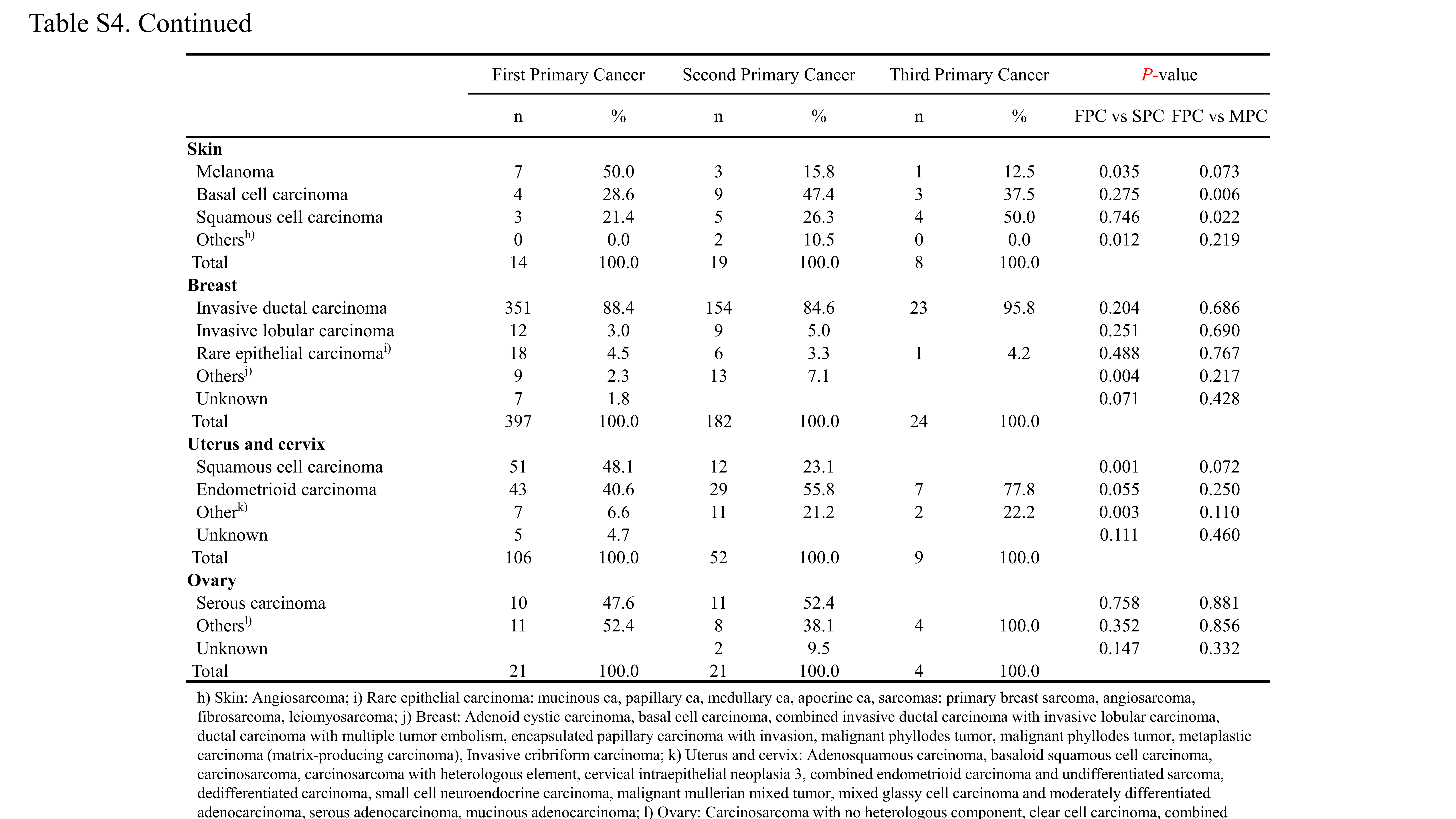

Supplement: Supplementary file 1 [file cancers-16-02346-s001.zip › Table S4-3.TIF]

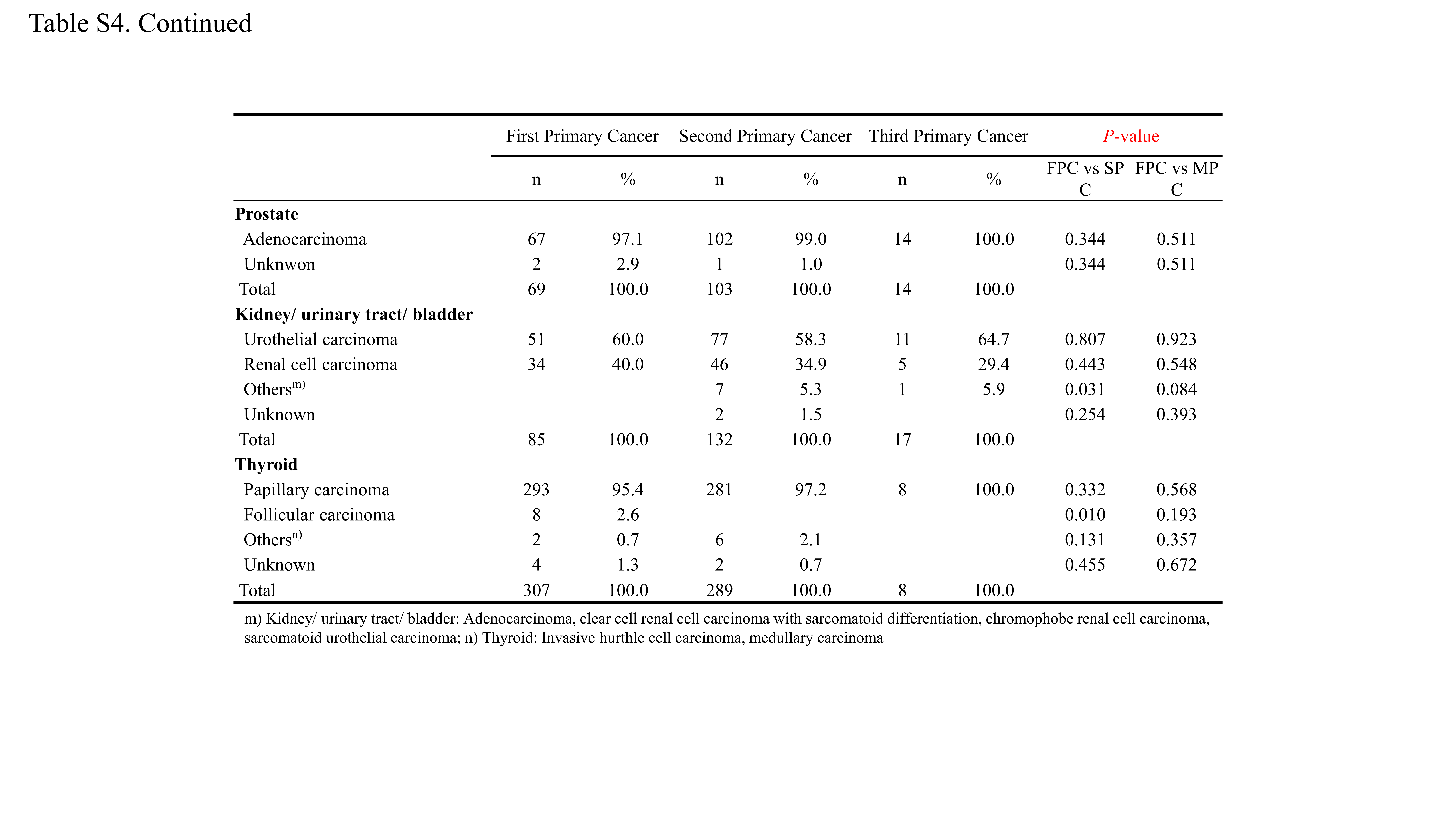

Supplement: Supplementary file 1 [file cancers-16-02346-s001.zip › Table S4-4.TIF]

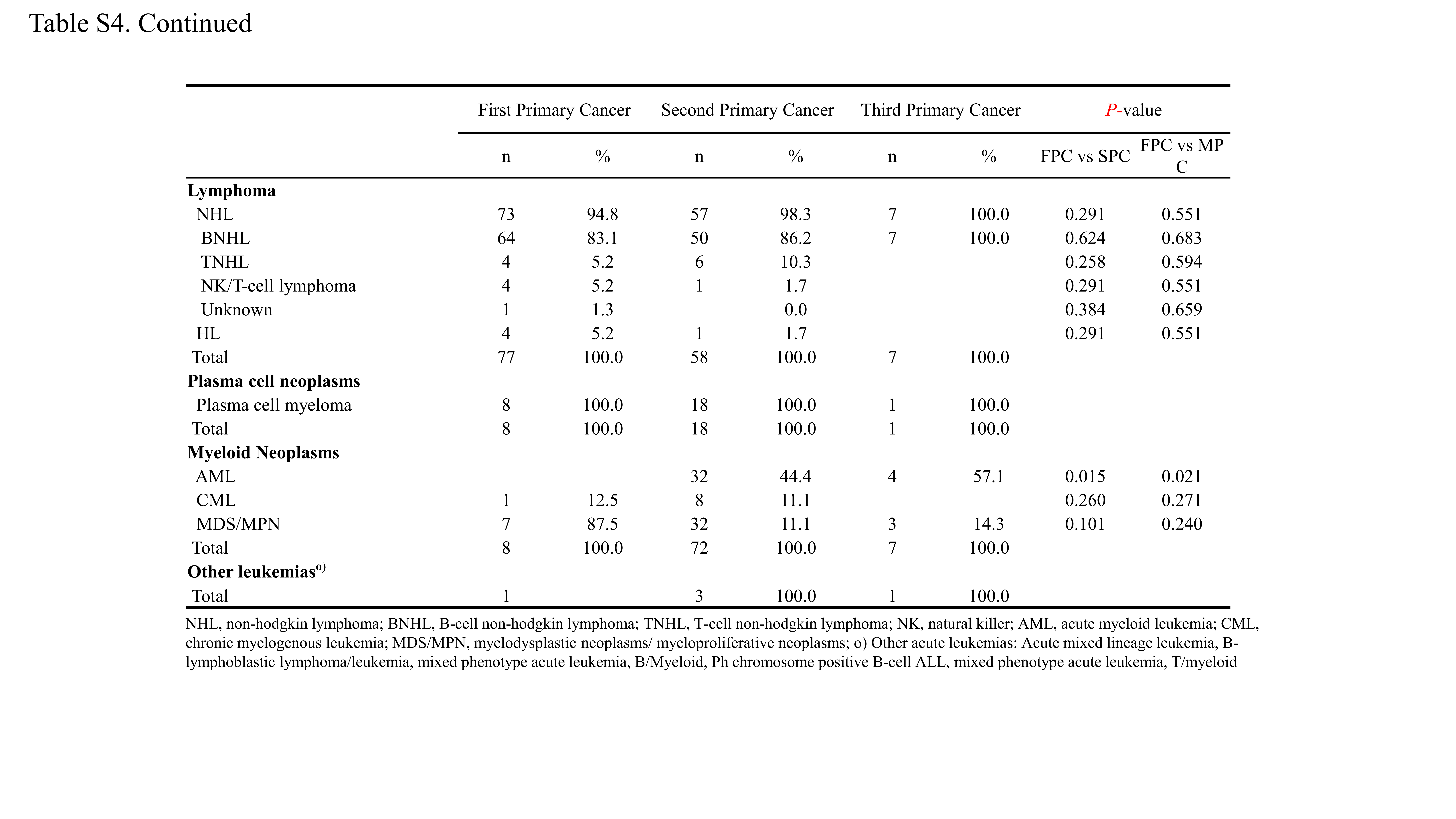

Supplement: Supplementary file 1 [file cancers-16-02346-s001.zip › Table S4-5.TIF]

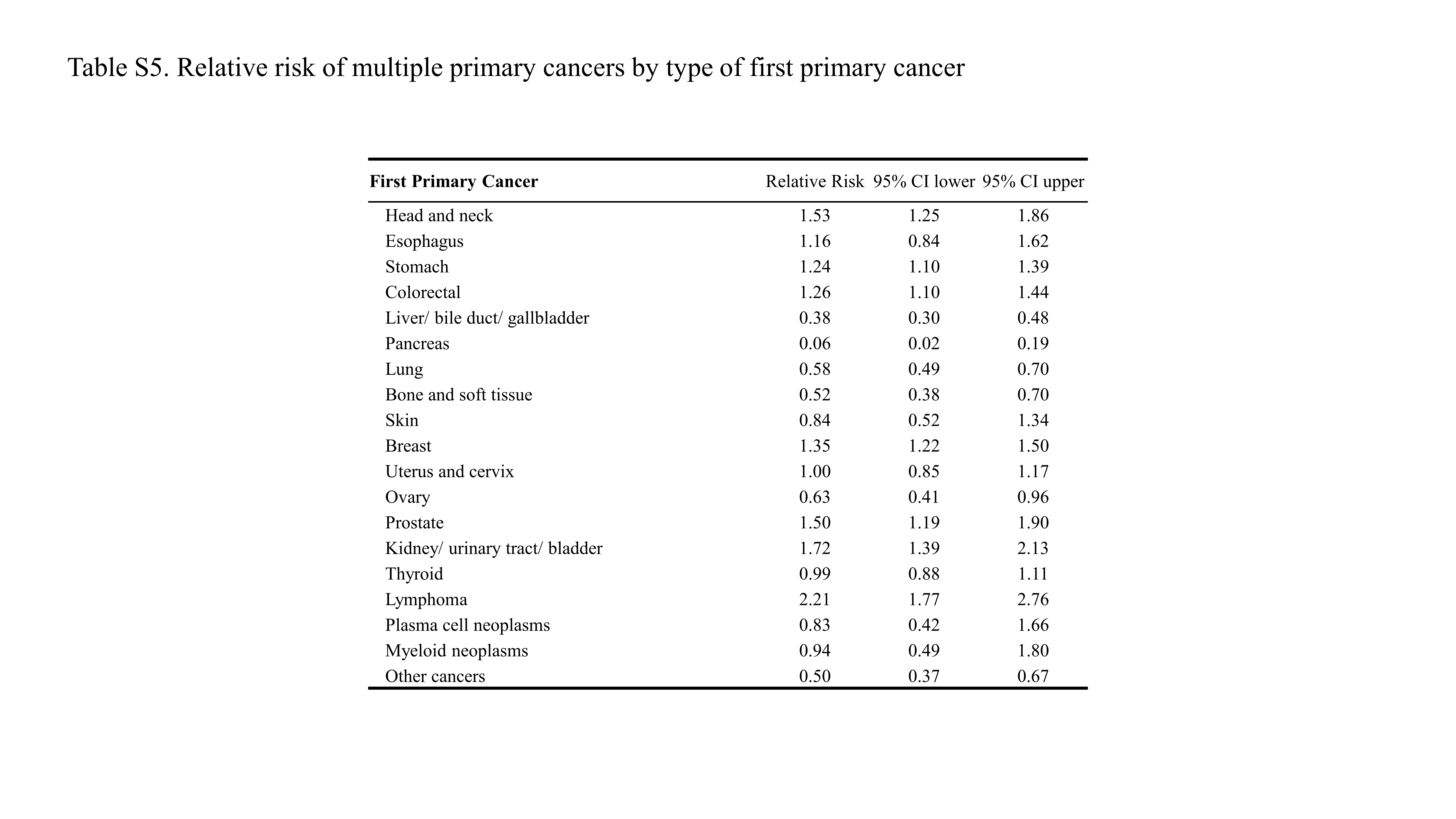

Supplement: Supplementary file 1 [file cancers-16-02346-s001.zip › Table S5.TIF]

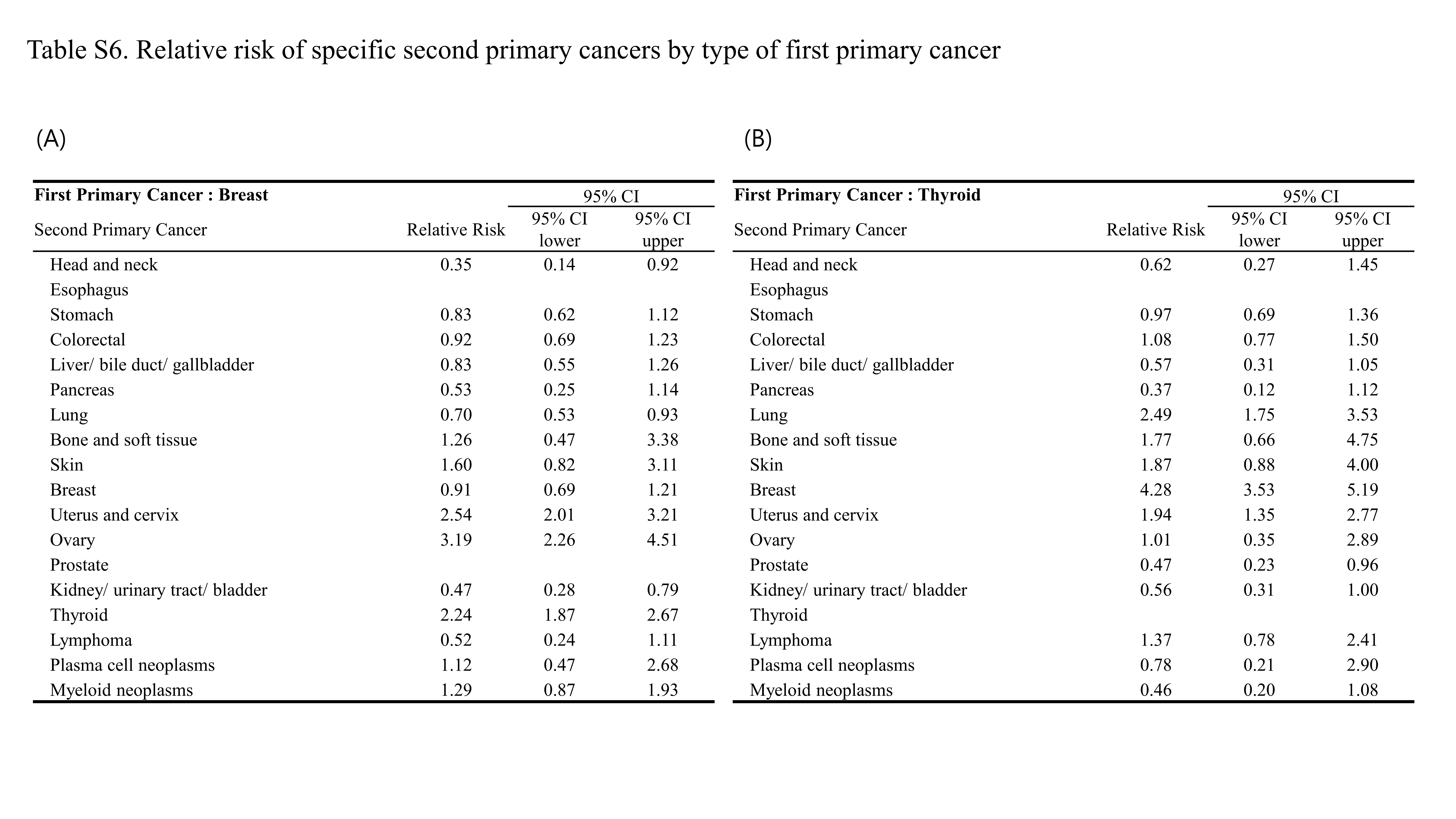

Supplement: Supplementary file 1 [file cancers-16-02346-s001.zip › Table S6-1.TIF]

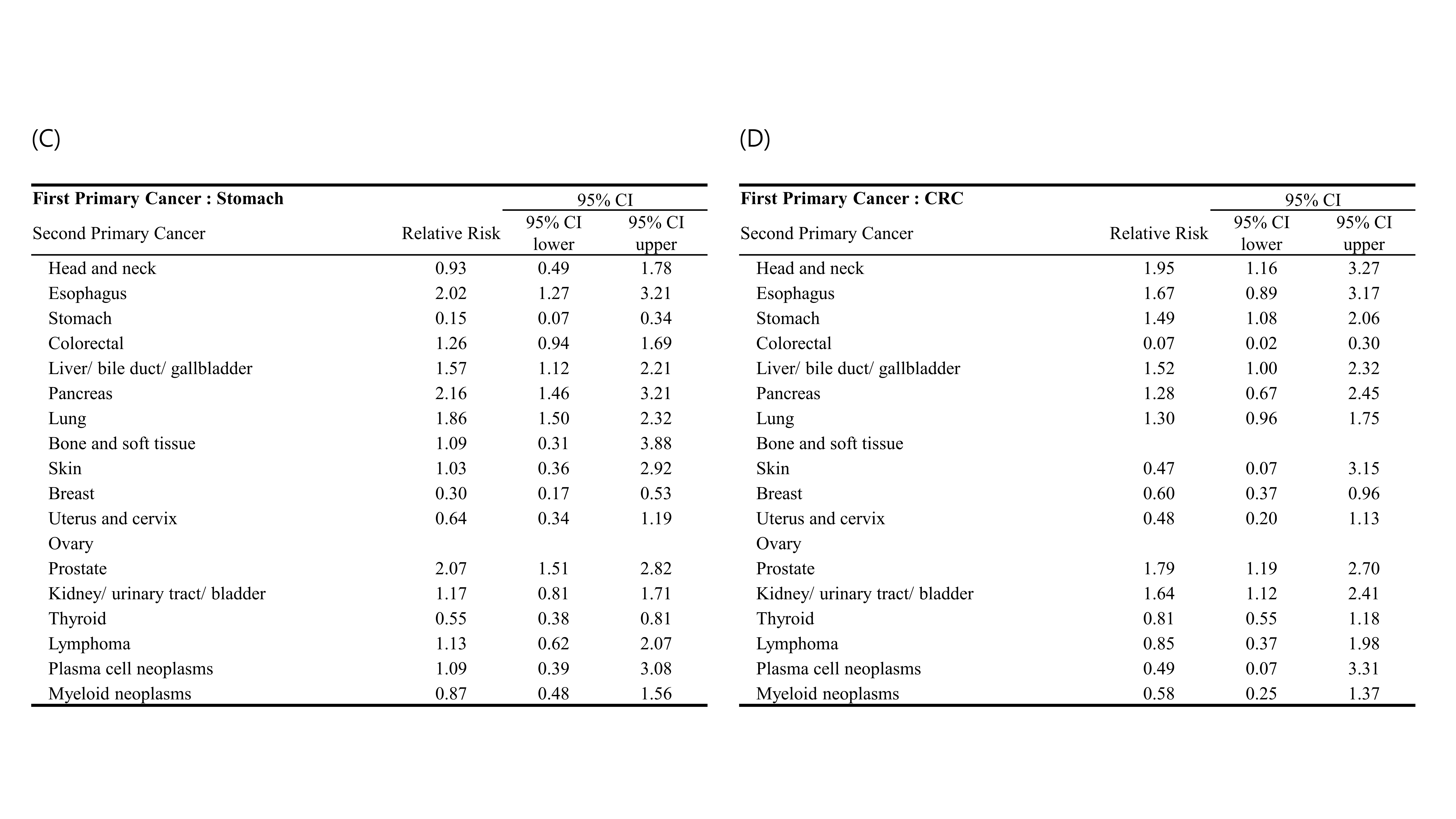

Supplement: Supplementary file 1 [file cancers-16-02346-s001.zip › Table S6-2.TIF]

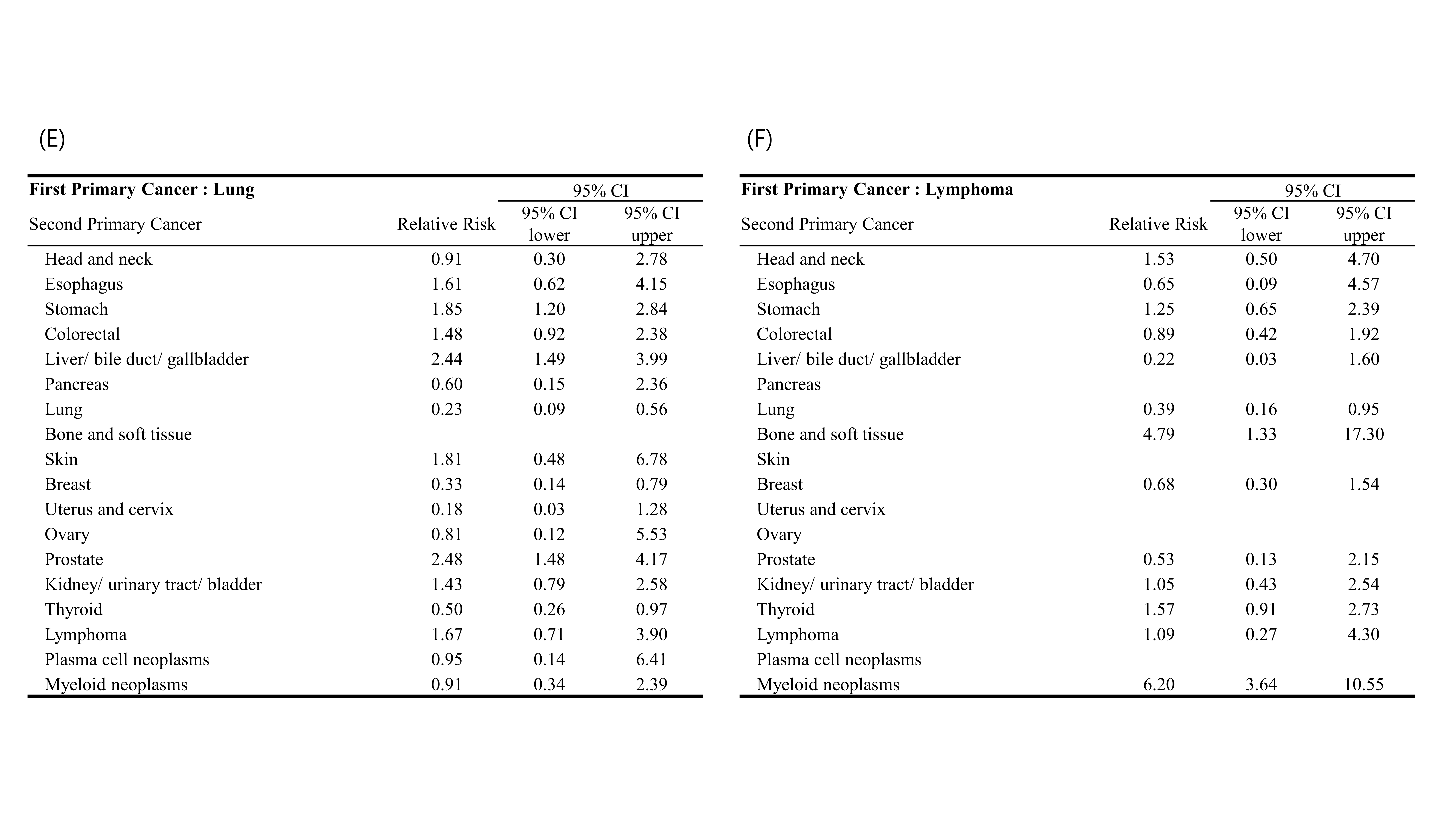

Supplement: Supplementary file 1 [file cancers-16-02346-s001.zip › Table S6-3.TIF]
